# Competing Theories on Global and Regional Vaccine Inequities: A Scoping Literature Review Within the Context of the COVID-19 Pandemic

**DOI:** 10.3390/vaccines13121254

**Published:** 2025-12-17

**Authors:** Karl Philipp Puchner, Elias Kondilis, Nasia Palantza, Stergios Seretis, Stavros Mavroudeas, Alexis Benos, Dimitris Papamichail

**Affiliations:** 1 Laboratory of Primary Health Care, General Medicine and Health Services Research, School of Medicine, Aristotle University of Thessaloniki, 540 06 Thessaloniki, Greece; karl.puchner@gmx.de (K.P.P.); ekondilis@auth.gr (E.K.); abenos@gmail.com (A.B.); 2 Department of Social Policy, Panteion University of Social and Political Sciences, 176 71 Athens, Greece; s.mavroudeas@panteion.gr; 3 Laboratory of Epidemiology, Health Determinants and Well-Being, Department of Public Health Policy, University of West Attica, 115 21 Athens, Greece

**Keywords:** global vaccine inequities, regional vaccine inequities, vaccine nationalism, vaccine apartheid, vaccine imperialism, vaccine diplomacy

## Abstract

**Background/Objectives**: Despite global efforts, COVID-19 revealed severe spatial vaccine inequities, disproportionately affecting low- and middle-income countries (LMICs). Scholars across disciplines proposed numerous—and often competing—terms and theories to explain these disparities. In this review and within the context of the COVID-19 pandemic, we assess the usage, definition, and appropriateness of these terms and their linked theories or frameworks. **Methods**: We conducted a scoping review aiming to clarify key definitions, concepts, and frameworks of eight prominent terms used in the literature regarding COVID-19 global and/or regional vaccine inequities (i.e., *vaccine nationalism*, *vaccine apartheid*, *vaccine colonialism*, *vaccine imperialism*, *vaccine racism*, *vaccine diplomacy*, *vaccine solidarity*, and *vaccine internationalism*). The methodology followed the Preferred Reporting Items for Systematic Reviews and Meta-analysis guidelines for Scoping Reviews and included papers from January 2020 to the end of October 2024. **Results**: We included 79 papers in our study. The majority (71%) were published in 2021–2022, with less than one-quarter authored by scholars from LMICs. *Vaccine imperialism* was consistently defined but rarely used, while *vaccine nationalism* and *vaccine apartheid* appeared more frequently with varied meanings. Yet, in most cases, all of these concepts identified economic interests of vaccine-producing countries as the root cause of the observed vaccine inequities. *Vaccine diplomacy* showed similar ambiguity, viewed by some as worsening inequities and by others as potentially mitigating them. The terms *vaccine racism*, *vaccine colonialism*, and *vaccine solidarity* were not explicitly identified but appear to be embedded within the definitions of other prominent terms detected. **Conclusions**: Across the preselected terms examined, we found numerous—and often conflicting—definitions, revealing the fragmented and competing understandings of the major drivers fueling global vaccine inequities. This lack of coherence inhibits evidence synthesis or shared theoretical progress but, most importantly, might undermine current and future efforts to address these inequities.

## 1. Introduction

Vaccine inequalities constitute a long-standing challenge of global health. Despite the large-scale production and the existence of well-established delivery systems—such as the national vaccination programs—vaccine inequalities continue to hinder the progress in controlling vaccine preventable diseases (VPDs) worldwide [1]. In line with J.T. Hart’s “inverse care law” [2], which states that, especially in privatized contexts, “the availability of good medical care tends to be inversely associated with the need of it in the population served”, vaccine inequalities result in lower immunization coverage among low- and middle-income countries (LMICs) and disadvantaged social groups within countries [3,4,5,6,7]. This, in turn, contributes substantially to the ongoing uneven global burden of VPDs [8].

COVID-19, an airborne infectious disease, has led to the most serious pandemic of the 21st century [9]. Alongside the unprecedented global efforts to develop and approve effective vaccines, the international health community also undertook significant measures to prevent inequitable access to these novel technologies. This is best exemplified by the establishment of the COVAX (COVID-19 Vaccines Global Access) platform, designed to accelerate the development and production of COVID-19 vaccines while, at the same time, theoretically aiming to ensure their fair and equitable distribution worldwide [10]. However, COVAX has faced substantial and well-founded scholarly criticism for its inefficiency, lack of transparency, and overly cautious stance on patent rights and the interests of the pharmaceutical industry [11]. Moreover, a growing body of literature and data suggests that the COVID-19 pandemic has severely intensified the existing vaccine inequalities, with devastating consequences for global health [12,13]. It is estimated that disparities in vaccine access alone were responsible for a considerable proportion of excess mortality linked to COVID-19—reaching as many as 1.3 million deaths worldwide in 2021 alone [14]. Even in the second and third year of the pandemic, despite a massive scale-up in vaccine production, spatial vaccine inequities did not appear to diminish substantially. Notably, WHO data from December 2023 show that, while more affluent WHO regions—such as the Americas and the Western Pacific—had vaccination coverage rates of over 80% with at least one dose, less affluent regions that include numerous LMICs, such as the African (AFRO) and Eastern Mediterranean (EMRO) regions, reached coverage levels of only 39% and 60%, respectively. These data suggest that, although procurement disparities might have narrowed as global supply increased, significant unevenness in vaccine availability persisted across global and regional contexts [15]. This has prompted an extraordinary academic interest in the field of vaccine inequalities in the context of COVID-19, with scholars trying to identify its underlying mechanisms [16,17,18].

Evidence generated so far suggests that, at the national level, COVID-19 vaccine inequalities were subjective to intrinsic factors such as health literacy and vaccine hesitancy [19,20], health access of different social groups [21,22], vaccine wastage, effectiveness and responsiveness of vaccine delivery systems in place, and the overall health system performance [23,24]. However, many researchers have argued that the root causes at the global and regional levels—which ultimately drive macro-level vaccine inequalities and are responsible for a deepening of the pre-existing global North—South divide in terms of vaccine distribution, accessibility and unmet demand —are fundamentally different [25,26,27,28]. Broader determinants—such as macroeconomic conditions, political and biomedical ethics, intellectual property rights, international trade agreements, biotechnology capabilities, production capacity, and geopolitical dynamics—seem to have played a significant role in shaping COVID19 vaccine access and unequal availability [19,29,30,31].

In this light, scholars from a wide array of disciplines have applied numerous theoretical frameworks to analyze and explain the phenomenon of vaccine inequality at the global and regional levels [32,33,34,35]. While this vast proliferation of competing theories reflects a vigorous and diverse research interest, which enhances interdisciplinary understanding of the phenomenon, it poses, at the same time, challenges for standardizing research in this field and complicates efforts to build academic consensus and draw generalizable conclusions [29,36,37].

Given the broad consensus that COVID-19 may be only the first in a series of future pandemics in the current century [9] and in the aftermath of the negotiations on the WHO-led initiative on a global, legally binding pandemic agreement, the need for a coherent and comprehensive explanatory model of the phenomenon of vaccine inequities becomes increasingly urgent [38]. Furthermore, the failure of multilateral efforts, such as COVAX, to prevent further aggravation of the vaccine inequities during the last pandemic crisis [39], combined with the eroding effects of the unfolding withdrawal of major donors and stakeholders such as the USA from global health commitments [40,41], underscores the critical necessity for a thorough analysis and comprehension of the structural drivers of vaccine inequities for the formulation of feasible, effective, and sustainable countermeasures.

In our review, we attempt to thoroughly map the major competing theories on COVID-19 global and regional vaccine inequities emerging from 2020 onwards. Specifically, our objective is to assess the usage, definition, and appropriateness of eight prominent terms and their corresponding theories that have been used to interpret spatial vaccine inequities within the context of the COVID-19 pandemic.

## 2. Methods

We conducted a scoping review aiming to identify gaps and clarify key definitions, concepts, and contrasting frameworks used in the literature to theoretically approach the major public health issue of COVID-19 vaccine inequities at the global and regional levels [42]. Building on the previous scientific work of the authors [29] and deep insights into the existing literature, we have critically chosen to focus on terms that might (a) cause and/or aggravate (e.g., *vaccine imperialism*, *vaccine nationalism*), (b) aggravate and/or ameliorate (e.g., *vaccine diplomacy*), or (c) ameliorate (e.g., *vaccine solidarity*) vaccine spatial inequities. The eight terms are *vaccine nationalism*, *vaccine apartheid*, *vaccine colonialism*, *vaccine imperialism*, *vaccine racism*, *vaccine diplomacy*, *vaccine solidarity, and vaccine internationalism*. During the process of reviewing the articles found, we were open to including additional relevant explanatory terms.

The methodology for this scoping review followed the Preferred Reporting Items for Systematic Reviews and Meta-analysis guidelines for Scoping Reviews (PRISMA-ScR) [43]. A review protocol was developed but not registered.

### 2.1. Eligibility Criteria

All types of research papers, also including editorials, letters to the editors, commentaries, and viewpoints, published in peer-reviewed journals from January 2020 were eligible in our review. We excluded books and conference papers.

The critical inclusion criterion was the paper’s scope. We included only papers (a) whose primary aim was to discuss and analyze regional or global vaccine inequities during the COVID-19 pandemic and (b) that used and provided justification for the usage of at least one of the following eight terms: *vaccine nationalism*, *vaccine apartheid*, *vaccine colonialism*, *vaccine imperialism*, *vaccine racism*, *vaccine diplomacy*, *vaccine solidarity, and vaccine internationalism*.

Papers referring to non-COVID-19 vaccine inequities and studies, either discussing COVID-19 vaccine inequities within countries (e.g., papers on vaccine denial, hesitancy, or acceptance) or focusing on country case studies (e.g., a specific country’s vaccine diplomacy policies, specific country’s access to global vaccine market), were excluded from our scoping review since our aim was to depict conflicting theories on COVID-19 vaccine inequities at the global and regional levels.

We included only studies to which we had full text access and whose main text was written in English.

### 2.2. Information Sources

The literature search of published studies was conducted in PubMed and Scopus, capturing papers published both in medical and social sciences journals. The search for sources was conducted between 7 and 25 October 2024.

### 2.3. Search Strategy

The key search terms included the previously mentioned eight prominent terms (*vaccine nationalism*, *vaccine apartheid*, *vaccine colonialism*, *vaccine imperialism*, *vaccine racism*, *vaccine diplomacy*, *vaccine solidarity, and vaccine internationalism*) and concepts on COVID-19 vaccination and inequalities using the following words and phrases: COVID-19, SARS-CoV-2, COVID-19 vaccines, health inequities, healthcare disparities, inequalities, inequities, disparities, injustice, and unfairness. Searches on each database were conducted using similar search concepts. The final search strategy is attached in a separate document (see Supplementary Materials: Table S1).

### 2.4. Selection of Sources of Evidence

Search results were exported to R, where duplicate removal occurred. Two researchers (K.P.P., N.P.) independently screened titles and abstracts for inclusion, followed by full-text screening. Any discrepant evaluation was resolved through discussion between the two researchers; unresolved discrepancies were discussed with an independent additional author (E.K.) to resolve.

### 2.5. Data Charting Process

A data charting table was developed using Excel to determine the variables to be extracted. Data items were verified by all authors. Two researchers independently charted the data from the included studies and discussed their findings or disagreements with a third reviewer in order to establish consensus.

### 2.6. Data Items

We abstracted data on the publication’s basic information (title, authors, first and last author’s country affiliation, year, journal, doi) and data on the article’s type, aim, and method used. Regarding the eight predetermined terms, we critically collected the following information: term(s) used in the paper, new identified terms, and comprehensive definition of terms if it was provided. In the case of multiple terms used, we noted whether the authors used them interchangeably or if they distinguished one from another and which term they adopted. Another important theme that was extracted regarded rationale and evidence for the use of the relevant term to explain vaccine inequities. Finally, we thoroughly abstracted data referring to possible criticism of the term(s) used and to the policy recommendations.

### 2.7. Synthesis of Results

Due to the heterogeneity of the studies (qualitative, mixed-methods, cross-sectional, and cohort studies), we used a narrative approach to analyze the included studies by utilizing text and a summarizing table to synthesize and explain the findings.

## 3. Results

A total of 1174 studies were obtained from the PubMed and Scopus databases. After removing 67 duplicates, we screened 1107 titles and abstracts. Following an initial screening, 182 books, book chapters, and conference papers were excluded, and 815 records were deemed ineligible because of not meeting the inclusion criteria, leaving 110 publications for full-text screening. However, two publications could not be retrieved in full text, five were not in the English language, and 24 did not meet the eligibility criteria regarding the paper’s main scope, leaving 79 articles for our scoping review (see Appendix A: Table A1).

**Figure 1:**
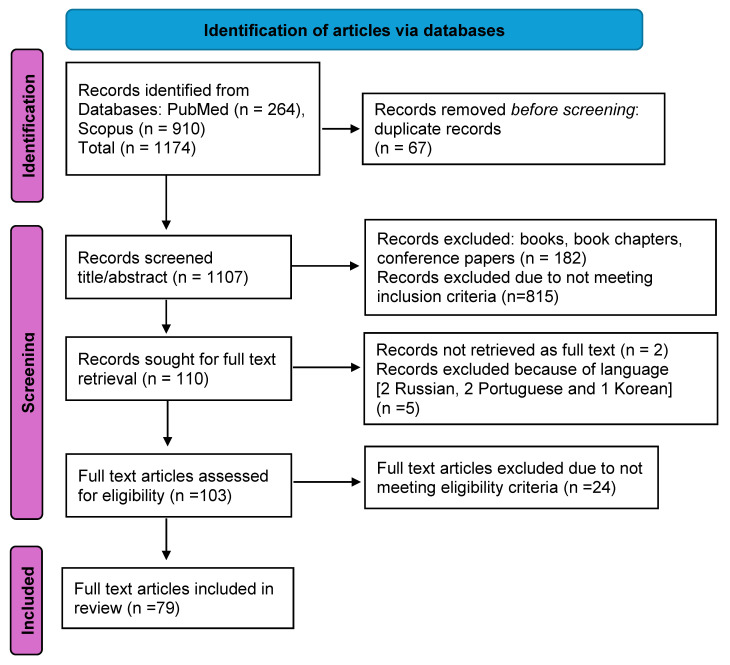
PRISMA-ScR flow diagram: selection of sources of evidence.

A PRISMA-ScR flow diagram describing the selection of sources of evidence is presented in Figure 1.

The majority (56/79) of the studies were published in 2021–2022, fourteen were published in 2023, eight were published in 2024, and only one was published in 2020. Recording first and last authors’ country affiliation, based on the World Bank country classifications by income level for 2024–2025, less than one-quarter (18/78) of the studies reviewed were authored by scholars from LMICs.

### Definitions of Terms and Corresponding Theories or Explanatory Frameworks (See Table 1)

The narrative analysis of the predetermined terms and their corresponding explanatory models yielded the following results. With respect to the terms describing factors that cause and/or aggravate vaccine inequities, *vaccine imperialism* exhibited a consistent definition throughout the literature included in our paper, being defined as the use of dominance and control by certain nations or blocs of nations over the research and development, production, distribution, and access to COVID-19 vaccines as a means for developed economies to exert power over and eventually exploit less developed economies, namely LMICs. *Vaccine imperialism* is thus understood to be a process of economic exploitation and subjugation of LMICs by patent-holding HICs and oligopolistic industries. By restricting and subordinating the global production and distribution of vaccines to economic interests, these actors not only significantly contribute to vaccine inequities but also to broader patterns of global economic injustice. However, it is important to note that the term vaccine imperialism appeared far less frequently than *vaccine nationalism* and *vaccine apartheid* in the papers reviewed.

With respect to *vaccine nationalism*, we identified four distinct definitions. Concretely, vaccine nationalism is understood by scholars as the tendency of HICs to (i) excessively purchase and hoard vaccines at the expense of LMICs; (ii) prioritize vaccination of their own general populations instead of addressing global needs, including the needs of vulnerable groups beyond their borders; and (iii) obstruct collective technology transfer efforts and patent waiver initiatives to protect national economic interests and national strategic pharmaceutic industries. In the fourth definition, encountered in one single publication, the term refers to a population’s preference for receiving a domestically developed and produced vaccine, coupled with a distrust of biomedical products from rival nations or those perceived as less developed. Typically, nationalism interprets disparities as the result of political will or ideology (“nation comes first” notion), while imperialism explains inequities as the result of economic exploitation and the alignment of policies to powerful industries’ interests. However, in the case of *vaccine nationalism* and across the first three definitions, the corresponding explanatory framework seems to be converging and overlapping—to some degree—with aspects of the *vaccine imperialism* term and theory [29]. Specifically, there is a broad consensus among scholars using the *vaccine nationalism* discourse that the main drivers of vaccine inequity are the competitive and patent-tied bilateral deals of HICs with pharmaceutical corporates, which, in turn, lead to limited availability of vaccines for the rest of the world, undermining multilateral purchasing initiatives such as COVAX and contributing to inflated global vaccine prices.

**Table 1:** Competing terms and theories on global and regional vaccine inequities.

	Definition	Corresponding Explanatory Framework	Comments	Bibliographical Examples *
Terms and theories explaining the causes and/or aggravation of vaccine inequities				
*Vaccine nationalism*	Defined as hoarding/stockpiling of vaccines by HICs.	The practice of HICs to purchase enough vaccines through advance market commitments and direct bilateral agreements with pharmaceuticals—at arbitrarily determined prices—to vaccinate their populations several times over and/or ban exports of key vaccine inputs at cost of global vaccine rollout of first dosage. This practice results in directly limited availability of and accessibility to vaccines for LMICs and weaking of multilateral purchase initiatives such as COVAX.	Often in the respective definition, the dimension of hoarding is coupled with export bans (of vaccines and/or critical inputs for vaccine production) and/or weakening of multilateral initiatives (directly with no participation for own purchase and indirectly through price inflation).	(Forman et al., 2023) (Wagner et al., 2021) (Lagman 2021)
	Defined as prioritization of vaccination of own country’s population over the global population.	Predominance of domestic obligations towards local constituency over international obligations in political ethics of HICs/Western democracies, leading to prioritization of vaccine procurement and stockpiling for own population—though not necessarily excluding the (lesser) engagement of governments in multilateral initiatives.	Often contrasted with the terms “vaccine cosmopolitanism”, “vaccine internationalism”, and “medical ethics”, which, according to the respective authors, stand for political practice and ethics where international/human-right obligations of governments prevail over national obligations. “Middle ground” political practice is considered in many of these papers as the most virtuous and feasible.	(Shao 2024) (Ferguson et al., 2020)
	Defined as the promotion of strategical economical national/corporate interests by vaccine-producing countries.	By blocking initiatives for TRIPS waiver, defending IP rights of (national) vaccine products, hindering knowledge exchange and production outsourcing in LMICs, and/or price reductions, HICs protect vital interests of (for them) strategically important pharma industries and use vaccine “scarcity” as a geopolitical tool.	In some papers, authors refer to “IP rights nationalism”, emphasizing the role of national governments in protecting IP rights of strategically important pharma industries and boycotting initiatives for issuance of TRIPS waivers and/or loosening of patent rights.	(Brown S et al., 2023) (Ho CM 2023) (Ba Z et al., 2024)
	Defined as a widespread sentiment in the general population of national pride/ownership and trust in vaccines produced either domestically and/or in a country considered “allied/alike”.	Trust and pride in a nationally produced vaccine and/or distrust and prejudice against vaccines manufactured in countries considered “foes”; “imperialistic” or “inferior” may influence, to some extent, vaccine acceptance and uptake in certain settings.	None of the included papers view “vaccine nationalism” exclusively under this lens; however, one paper discusses this additional dimension of the term.	(Vanderslott S et al., 2021)
*Vaccine apartheid*	Defined as the practice by high-income countries of purchasing and hoarding vaccines, ostensibly for domestic use, leading to limited availability for low- and middle-income countries (LMICs); this overlaps with one of the previous definitions of vaccine nationalism.	Institutional dysfunction and fragmentation within global health—exacerbated by the involvement of multiple stakeholders, such as pharmaceutical companies and philanthropic organizations—have contributed to a multi-tiered vaccine production and distribution system. This structure enables high-income countries (HICs) to purchase unlimited vaccine supplies, leaving many low- and middle-income countries (LMICs) marginalized in a manner resembling the ‘apartheid regime’.	Many authors suggest that this practice attributed to the emergence of novel COVID-19 variants in under-vaccinated geographic regions.	(Tumwine J 2021) (Brown S et al., 2023)
	Defined as the racialized distribution of vaccines or the practice of creating conditions of premature death, poverty, and disease in a racialized way.	Vaccine availability remains extremely limited in parts of the world referred to as ‘dark nations,’ privileging the lives of those in the Global North over those in the Global South. This disparity reflects a proportional relationship to countries’ income levels and is driven by racialized prioritization and distribution practices. These practices are rooted in longstanding institutional racism—embedded in policies, laws, and systems—that perpetuates segregation and discrimination against historically marginalized populations both within and across nations. In the context of global health, such mechanisms serve to establish and maintain the dominance of one racial group over others.	The term ‘apartheid’ has been introduced in the context of the COVID-19 pandemic by scholars who argue that it most accurately captures the complexity and root causes of vaccine inequalities. It highlights the perception and reality of differential rights afforded to distinct segments of the global population. For instance, many low- and middle-income countries (LMICs) served as Phase III clinical trial sites—contributing infrastructure and trial participants and thereby sharing the risks of research and development. However, these same countries were largely excluded from access to the resulting vaccines, illustrating a form of segregation where risks were shared but benefits were not.	(Sirleaf M 2022) (Forman L et al., 2023) (Lanziotti VS et al., 2022)
	Vaccine apartheid is understood as the limited availability and affordability of COVID-19 vaccines in LMICs and the restricted capacity of most of these countries to produce them.	This reflects the deliberate obstruction of technology transfer and local vaccine production in LMICs, stemming mainly from the strict enforcement of intellectual property rights (IPRs) by HICs.	Authors using this definition of the term seem to be using it somehow interchangeably with the term vaccine nationalism.	(Privor-Dumm L et al., 2023) (Fox AM et al., 2023) (Paquin S et al., 2023)
*Vaccine imperialism*	This refers to the exercise of disproportionate power and influence by certain nations or blocs over the global supply, distribution, and access to vaccines. It reflects a contemporary phenomenon in which advanced industrialized countries exert significant control over vaccine production, technology, and distribution channels—thereby perpetuating and deepening existing global health inequalities.	Vaccine imperialism is understood as the intersection of COVID-19 and imperialism, highlighting how vaccines—and pharmaceuticals more broadly—function as instruments of economic exploitation under the current global capitalist system. In this context, vaccines facilitate the transfer of value from user countries to those that hold patents and control production. Less-developed countries, dependent on imperialist economies for the development, manufacturing, and distribution of these health technologies, are thus subject to systemic exploitation. Moreover, this exploitation is sustained by national and global governance structures that not only permit but actively reinforce not only vaccine inequalities but also economic injustices at the global level.	While all authors identify value transfer to the disadvantage of less industrialized countries as the central mechanism of vaccine imperialism, one paper further highlights a hegemonic worldview in which the Global North—as the epicenter of capitalism—is assigned a higher ‘value of statistical life’.	(Seretis SA et al., 2024) (Fallah MP et al., 2022)
*Vaccine racism*	The term was not encountered in our scoping literature review as such but seems to be incorporated into one of the previously mentioned definitions of vaccine apartheid.			
*Vaccine colonialism*	The term was not encountered in our scoping literature review but seems to be incorporated into the definition of vaccine neocolonialism.			
*Vaccine neocolonialism* *[new similar identified term]*	The unequal distribution, rollout, and uptake of COVID-19 vaccines worldwide, especially in low- and middle-income countries (LMICs) that have been historically plagued by colonialism.	Power structures within the WHO and other international agencies continue to reflect a colonial ‘world order,’ prioritizing approaches and policies that favor the Global North over the Global South. Additionally, high-income countries (HICs) refused to agree to the TRIPS waiver mechanism, which would have suspended intellectual property protections for COVID-19 vaccine-related research and development. Both factors can be understood as forms of neocolonialism contributing to the observed vaccine inequalities.	The term is sometimes used interchangeably with ‘vaccine apartheid,’ as noted in one paper that introduced the concept.	(Shah DM et al., 2022)
Terms and theories explaining both the cause/aggravation and amelioration of vaccine inequities				
*Vaccine diplomacy*	Defined as collaboration, infrastructure development, and knowledge sharing between actors from different countries—whether governmental or non-governmental—with the aim of developing, producing, and licensing novel COVID-19 vaccines across multiple nations. This is often cited as an example of medical or public health diplomacy.	Within the context of medical and public health diplomacy led by biomedically advanced countries, vaccines are donated, multilateral purchasing mechanisms—such as COVAX—are established, and infrastructure and knowledge are shared, all contributing to narrowing the North–South vaccination gap.	Sometimes also referred to as vaccine science diplomacy, which involves the joint efforts of scientists from two or more nations—despite geopolitical or ideological rivalries—to develop new vaccines. However, some authors note that vaccine diplomacy can carry an implicit prioritization of home and allied countries first.	(Varshney S et al., 2021) (Horng DC 2024) (Amankwah-Amoah J et al., 2022)
	Defined as a nation’s vaccine efforts aimed at building mutually beneficial relationships with other countries through the use of vaccines and their distribution as tools of diplomacy, soft power, and foreign policy.	Bilateral donations or purchases may bypass multilateral procurement efforts. Also, vaccine diplomacy is focusing solely on vaccine donations without facilitating knowledge transfer, thereby exacerbating vaccine inequalities long-term.	Some authors also highlight the negative impact vaccine diplomacy can have on immunization efforts within the donor country (e.g., Russia), where large quantities of domestically produced vaccines may be allocated for export or donation, potentially limiting availability for the local population.	(Brown S et al., 2023) (de Bengy Puyvallée A et al., 2022)
	Non-Western and emerging powers widely distributed vaccines and promoted them as public goods, thereby shifting the market dynamics surrounding COVID-19 vaccines.	Emerging vaccine manufacturing powers are not only more likely to donate vaccines through bilateral deals as part of vaccine diplomacy but are also more inclined to share technology, thereby stimulating production in other LMICs and fostering robust, sustainable vaccine manufacturing. All of these factors contribute positively to reducing vaccine inequalities.	Although this definition overlaps somewhat with previous ones, most authors emphasize that vaccine diplomacy, in parallel to serving as a soft power tool for advancing the foreign policy goals of the countries engaging in it, can substantially challenge the oligopolistic practices of Western high-income countries.	(Suzuki M et al., 2023) (Liu L et al., 2022) (Apolinario L et al., 2022)
Terms and theories explaining the amelioration of vaccine inequities				
*Vaccine internationalism*	Used as the exact opposite to vaccine nationalism, implying coordinated action from multiple stakeholders for overcoming vaccine shortages at the regional and/or global level.	The authors using this term refer to the historic precedent of coordinated compulsory licensing of HIV drugs by South Africa, India, and Brazil, arguing that similar collective action could help reduce global COVID-19 vaccine inequalities.	The term is found only in one of the included papers.	(Banerjee D 2021)
*Vaccine solidarity*	The term was not encountered in our scoping literature review.			
*Vaccine empathy* *[new similar identified term]*	The donation of vaccines from one country—typically more affluent—to another, usually less wealthy, reflecting a nation’s capacity to empathize with the needs and desires of others.	While acts of vaccine empathy have been observed, their unsustainability largely explains, according to the authors, the persistence of global vaccine inequities.	Some authors regard vaccine empathy as the most effective way to address vaccination gaps promptly, since measures like waiving patents or negotiating vaccine diplomacy deals tend to be time-consuming.	(Su Z et al., 2021) (Hotez PJ 2022)
*Vaccine cosmopolitanism* *[new similar identified term]*	A view of distributive justice for vaccines that holds community membership—such as citizenship or national belonging—as ethically irrelevant.	Vaccine cosmopolitanism is regarded as a means to reduce vaccine inequities by ensuring needs-based distribution and prioritizing the most vulnerable populations at the global level.	In this context, the term is used by the authors as the complete opposite of ‘vaccine nationalism,’ with some arguing that both extremes—when taken to their radical forms—are utopian or dystopian and, therefore, unrealistic or unfeasible.	(Emanuel EJ et al., 2021) (Collste G 2022)

* See Appendix A: Table A1.

The definitions of *vaccine apartheid* showed considerable variation in the papers included in our study. While two definitions and their corresponding explanatory frameworks closely resembled aspects of *vaccine nationalism* and were even used interchangeably with that term in the respective texts, another definition defined *vaccine apartheid* as the racialization of COVID-19 vaccine distribution and access. As such, this definition of *vaccine apartheid* seems to incorporate the concept of *vaccine racism*, a term that was not identified in our scoping review as such. Similarly, the term *vaccine colonialism* did not emerge in our review; however, a closely related term, *vaccine neocolonialism*, was identified in one publication, where the authors noted that it is sometimes used interchangeably with *vaccine apartheid*, and refers to the unfair global distribution of vaccines due to global governance structures still carrying their colonial heritage and colonial uneven power distribution.

In the case of *vaccine diplomacy*—a term related by scholars to both aggravation and amelioration of vaccine inequalities—we were able to identify three distinct definitions in the literature analyzed. While some authors define *vaccine diplomacy* as a subset of medical and public health diplomacy, i.e., the mutually beneficial collaboration of nations and unrestricted knowledge transfer aiming at the development of globally available and affordable biomedical technologies, others focus on how certain powers subordinate national vaccine production and purchase and distribute/donate to serve broader geopolitical and diplomatic agendas. In addition, some scholars identify *vaccine diplomacy* as a particular soft power tool used by non-Western emerging global actors such as India and China, with the intention of reducing global dependency on Western vaccine research and development and, thereby, contributing to the diversification of the global COVID-19 vaccine market. Irrespective of the definition adopted, all authors—albeit with varying emphases—acknowledge that *vaccine diplomacy* can yield mixed results in terms of equitable access to the COVID-19 vaccines, converging rather on the inherent ambiguity of this approach as a means for crucially addressing vaccine inequities in the context of the COVID-19 pandemic.

Finally, the predetermined terms referring to factors ameliorating vaccine inequities were identifiable only in a small number of papers fulfilling our inclusion criteria. Vaccine internationalism was defined by D. Banerjee as the coordinated action from multiple stakeholders to overcome vaccine shortages at the regional and/or global level—an approach rooted in the historical precedent of compulsory licensing of HIV drugs by South Africa, India, and Brazil and standing, according to the author, in stark contrast to the term vaccine nationalism. In addition, though no paper using the term vaccine solidarity was identified in our review, two closely related concepts, i.e., vaccine empathy and vaccine cosmopolitanism, were sporadically encountered in the literature analyzed. Both terms, though not identical, imply practices and approaches transcending national interests and boarders, prioritizing global vaccine equity, with some of the authors employing them, critically examining their feasibility and sustainability.

## 4. Discussion

To our knowledge, this is the first scoping review to examine a substantial number of terms (with their definitions and corresponding theories) that were used as interpretation tools for the observed vaccine inequities during the COVID-19 pandemic. Our approach enabled us to capture the efforts of scholars across disciplines to understand and analyze vaccine inequities at both regional and global levels, revealing at the same time their competing interpretations and, therefore, competing proposed solutions to mitigate these inequities.

Our analysis exhibits a notable limitation. Since our objective was to map the major competing theories on COVID-19 vaccine inequities and not to focus on answering a specific research question regarding one explanatory term, we did not perform a systematic review. We also restricted our scoping review to the PubMed and Scopus databases, and we only included articles written in English. All the above choices may have led to the omission of some critical terms—such as vaccine solidarity—and theories regarding COVID-19 vaccine inequities and the failure to cite important studies, most probably emerging from the Global South and/or being written in other world languages (e.g., Spanish, French, or Arabic). In addition, by limiting ourselves to only two databases—of which one has an almost exclusively biomedical scope (i.e., Pubmed)—we may have partially compromised the likelihood of identifying highly relevant articles originating from important non-biomedical disciplines, such as political science, political economy, philosophy, or sociology. Furthermore, we did not conduct a critical appraisal of individual sources of evidence. It is worth noting that less than one-fourth of the papers included in our study originated from academic institutions of LMICs, with the overwhelming majority being generated in HICs. This underrepresentation, likely due to persistent publication bias favoring HICs or disparities in research capacity between LMICs and HICs, likely hinders our understanding of the phenomenon related to COVID-19 vaccine inequities, as theories emerging from regions most directly affected by the causes and consequences of this inequity seem to be scarce, at least in the dominant academic literature. Thus, in total, the aforementioned limitations may have, to some extent, biased our results by underrepresenting certain geographic regions and disciplinary perspectives as well as by potentially excluding critical discourses embedded in non-Anglophone academic traditions and contexts.

Taking this limitation into account, our analysis was able to find a large variety of—often overlapping and in some cases conflicting—definitions and explanatory theories regarding global and regional vaccine inequities. This variation in some cases seems to be subject to the geographic affiliation of scholars using a specific term or to their disciplinary background.

For example, some authors affiliated to HICs’ institutions tend to give to the term *vaccine diplomacy* a far more negative connotation compared to authors coming from vaccine-producing MICs. The first group of authors argues that bilateral *vaccine diplomacy* bypasses more transparent multilateral mechanisms—such as COVAX—compromising global allocation efforts, and risks unmet vaccination needs at the national level if practiced at a large scale [11,44]. Contrary to this view, most authors from MICs involved in *vaccine diplomacy* activities view this term in a far more positive way, as they regard it not only as a means of immediate amelioration of global vaccine inequity but also as an essential instrument for diversifying and reshaping the global vaccine market in favor of LMICs, including the transfer of knowledge for local vaccine production [45,46]. Of course, both groups of authors acknowledge *vaccine diplomacy* as a soft power political tool employed by individual countries to serve, among others, their national interests and its complementary-only role to multilateral initiatives for addressing vaccine inequities at a global level.

Another example is scholars’ differential perspectives, depending on their discipline, regarding the terms and concepts of *vaccine internationalism*, *vaccine empathy*, and *vaccine cosmopolitanism*. All authors use these terms, although distinct from each other, in an interchangeable and overlapping manner, typically referring to practices that ameliorate vaccine inequities, and as such, these terms were used in all cases as a contrast to *vaccine nationalism*. However, authors with a biomedical background are likely to urge for full policy adoption of these approaches, while scholars from humanities and social sciences, while praising these practices as ethical and potentially effective means of combating vaccine inequities, tend at the same time to critically elaborate on the moral and political viability of these concepts within the context of current sociopolitical realities [47,48]. Some of these scholars even seem to be defending a form of moderate vaccine nationalism as a pragmatic, middle-ground approach in a world marked by competing obligations [49].

Another striking finding of our review is the ambiguity regarding the most frequently used terms in the literature, namely *vaccine nationalism* and *vaccine apartheid*, exhibiting multiple definitions [50,51]. In many cases, vaccine nationalism and vaccine apartheid are used as economic rather than political concepts (e.g., “nation comes first” or “racial discrimination” notions), assessing the exploitation and subordination of LMICs by vaccine-producing and patent-holding HICs. This interpretation closely aligns with—though seldom names—the broader theory of vaccine imperialism [29]. On the contrary, vaccine imperialism, limited in its use, shows notable consistency in definition and corresponding theory, with authors originating from both LMICs and HICs using this term to provide a comprehensive and realistic analysis of the causal association between economic imperialism and the observed vaccine spatial inequities during the pandemic.

Understanding and overcoming the fragmented and competing interpretations of the drivers of global vaccine inequities, as demonstrated in our paper, are not merely a theoretical academic exercise but have far reaching global health policy and governance implications [33]. The emergence of the next pandemic is widely regarded as a matter of when not if [9]. This public health threat, combined with the massive failure of recent multilateral mechanisms—such as COVAX—to prevent vaccine inequities during the COVID-19 pandemic [52], highlights the urgency of the issue. Additionally, the global health landscape is undergoing rapid change, marked by the withdrawal of major actors such as the United States from joint global health efforts [53]. In this context, it is of utmost importance to identify scientifically appropriate terms, definitions, and frameworks that can support transdisciplinary, in-depth analysis of this complex public health challenge [54]. In-depth analysis must offer insights that can inform cross-sectoral policies and guide structural reforms within the global health architecture to strengthen future pandemic preparedness and response at the global level [54]. Our scoping review represents an initial step in mapping the conflicting theories and terms used in research on vaccine inequity in the context of COVID-19. Future work should build on our findings by engaging in an interdisciplinary, LMIC-inclusive, consensus-building process to select the most scientifically appropriate terms and to standardize analytical frameworks, thereby enabling the in-depth analysis urgently needed to better understand vaccine inequity during the COVID-19 pandemic.

## 5. Conclusions

Despite the consensus that global and regional vaccine inequities were exacerbated during the COVID-19 pandemic, terms and theories capturing and interpreting this significantly negative public health development remain ambiguous and conflicting. Our scoping review identified at least five terms/theories (*vaccine nationalism*, *apartheid*, *imperialism*, *neo-colonialism*, and *diplomacy*) and multiple differential definitions within each term/theory, trying to explain the failure of not achieving global, equitable vaccine coverage and roll-out during the recent pandemic crisis. Our scoping review also found that vaccine nationalism and vaccine apartheid are the terms most often used to describe spatial vaccine inequities. In many cases, they frame these inequities as forms of power subordination and economic exploitation of LMICs by vaccine-producing and patent-holding HICs. Although seldom named explicitly, this perspective closely aligns with the broader discourse of vaccine imperialism.

The fragmented and conflicting understanding of the drivers of global vaccine inequities obviously reflects the political tensions, conflicting interests, and different approaches on how these inequities should be addressed. The recent WHO pandemic agreement is a typical example; for many, it represents a major step in the right direction [55], but for others—due to its voluntary provisions on vaccine technology transfers and the limited protection it offers to LMICs against trade disputes when exercising their rights for compulsory licensing [56], i.e., the lift of patents’ frame due to public health emergencies—it is an incomplete step that risks repeating past failures.

## Data Availability

The original contributions presented in this study are included in the article/Appendix A/Supplementary Material. Further inquiries can be directed to the corresponding author.

## Supplementary Materials

The following supporting information can be downloaded at: https://www.mdpi.com/article/10.3390/vaccines13121254/s1, Table S1. PubMed and Scopus search strategy.

## Abbreviations

The following abbreviations are used in this manuscript:

LMICs	Low- and Middle-Income Countries
VPDs	Vaccine Preventable Diseases
COVAX	COVID-19 Vaccines Global Access

## Appendix A

**Table A1:** Articles included in the scoping review and their basic characteristics (n = 79).

AA	Authors	Publication Year	Country Affiliation (First/Last Author)	Country’s Affiliation Income Level * (First and Last Author)	Title	DOI or PMC or Link
1	Adekola TA et al.	2023	Australia/Canada	High-income economies (both)	Human rights law, intellectual property and vaccine nationalism: lessons for the post-COVID-19 world	10.1080/1323238X.2023.2286941
2	Ahlberg BM et al.	2022	Sweden/Sweden	High-income economies (both)	Ethnic, racial and regional inequalities in access to COVID-19 vaccine, testing and hospitalization: Implications for eradication of the pandemic	10.3389/fsoc.2022.809090
3	Apolinario L et al.	2022	Brasil/Chile	Mixed	Chinese and Indian COVID-19 Vaccine Diplomacy during the Health Emergency Crisis	10.1590/0034-7329202200114
4	Amankwah-Amoah J	2022	UK	High-income economies (both)	COVID-19 and counterfeit vaccines: Global implications, new challenges and opportunities	10.1016/j.hlpt.2022.100630
5	Amankwah-Amoah J et al.	2022	UK/Ghana	Mixed	COVID-19 pandemic, vaccine nationalism and counterfeit products: Discourse and emerging research themes	10.1002/tie.22302
6	Ashindorbe K et al.	2024	Nigeria/Nigeria	Middle- and low-income economies (both)	Vaccine nationalism and the quest for indigenous COVID-19 vaccine in Nigeria	10.1080/14725843.2022.2034597
7	Avdagic S et al.	2024	UK/UK	High-income economies (both)	Issue framing, political identities, and public support for multilateral vaccine cooperation during COVID-19	10.1111/1475-6765.12628
8	Ba Z et al.	2024	China/China	Middle- and low-income economies (both)	Vaccine inequity-induced COVID-19 dilemma: Time to sober up	10.1016/j.legalmed.2023.102364
9	Bajaj SS et al.	2022	USA/USA	High-income economies (both)	Vaccine apartheid: global cooperation and equity	10.1016/S0140-6736(22)00328-2
10	Banerjee D	2021	USA	High-income economies (both)	From Internationalism to Nationalism A New Vaccine Apartheid	10.1215/1089201X-9407806
11	Beaton E et al.	2021	UK/USA	High-income economies (both)	Crisis Nationalism: To What Degree Is National Partiality Justifiable during a Global Pandemic?	10.1007/s10677-021-10160-0
12	Bhattacharya S et al.	2021	India/India	Middle- and low-income economies (both)	Role of vaccine science diplomacy in low-middle-income countries for eradicating the vaccine-preventable diseases: Targeting the “LAST MILE”	10.4103/jfmpc.jfmpc_2253_20
13	Binagwaho A et al.	2022	Rwanda/USA	Mixed	Equitable and Effective Distribution of the COVID-19 Vaccines—A Scientific and Moral Obligation	10.34172/ijhpm.2021.49
14	Bolcato M et al.	2021	Italy/Italy	High-income economies (both)	COVID-19 Pandemic and Equal Access to Vaccines	10.3390/vaccines9060538
15	Borges LC et al.	2022	USA/USA	High-income economies (both)	More Pain, More Gain! The Delivery of COVID-19 Vaccines and the Pharmaceutical Industry’s Role in Widening the Access Gap	10.34172/ijhpm.2022.6942
16	Brown S et al.	2023	Canada/Canada	High-income economies (both)	COVID-19 vaccine apartheid and the failure of global cooperation	10.1177/13691481231178248
17	Chatterjee N et al.	2021	Norway/Sweden	High-income economies (both)	Politics of Vaccine Nationalism in India: Global and Domestic Implications	10.1080/08039410.2021.1918238
18	Collste G	2022	Sweden	High-income economies (both)	‘Where you live should not determine whether you live’. Global justice and the distribution of COVID-19 vaccines	10.1080/16544951.2022.2075137
19	de Bengy Puyvallée A et al.	2022	Norway/Norway	High-income economies (both)	COVAX, vaccine donations and the politics of global vaccine inequity	10.1186/s12992-022-00801-z
20	Duan Y et al.	2021	China/China	Middle- and low-income economies (both)	Disparities in COVID-19 Vaccination among Low-, Middle-, and High-Income Countries: The Mediating Role of Vaccination Policy	10.3390/vaccines9080905
21	Dube K	2022	South Africa	Middle- and low-income economies (both)	COVID-19 vaccine-induced recovery and the implications of vaccine apartheid on the global tourism industry	10.1016/j.pce.2022.103140
22	Dzau VJ et al.	2022	USA/USA	High-income economies (both)	Closing the global vaccine equity gap: equitably distributed manufacturing	10.1016/S0140-6736(22)00793-0
23	Eccleston-Turner M et al.	2021	UK/UK	High-income economies (both)	International Collaboration to Ensure Equitable Access to Vaccines for COVID-19: The ACT-Accelerator and the COVAX Facility	10.1111/1468-0009.12503
24	Emanuel EJ et al.	2021	USA/USA	High-income economies (both)	On the Ethics of Vaccine Nationalism: The Case for the Fair Priority for Residents Framework	10.1017/s0892679421000514
25	Fajber K	2022	Canada	High-income economies (both)	Business as Usual? Centering Human Rights to Advance Global COVID-19 Vaccine Equity Through COVAX	PMC9790946
26	Fallah MP et al.	2022	Ethiopia/Canada	Mixed	When maximizing profit endangers our humanity: vaccines and the enduring legacy of colonialism during the COVID-19 pandemic	10.1080/07078552.2022.2047475
27	Ferguson K et al.	2020	USA/USA	High-income economies (both)	Love thy neighbour? Allocating vaccines in a world of competing obligations	10.1136/medethics-2020-106887
28	Ferranna M	2024	USA	High-income economies (both)	Causes and costs of global COVID-19 vaccine inequity	10.1007/s00281-023-00998-0
29	Forman L et al.	2023	Canada/Canada	High-income economies (both)	Can we move beyond vaccine apartheid? Examining the determinants of the COVID-19 vaccine gap	10.1080/17441692.2023.2256822
30	Fox AM et al.	2023	USA/Hong Kong	High-income economies (both)	Substantial Disparities In COVID-19 Vaccine Uptake And Unmet Immunization Demand In Low-And Middle-Income Countries	10.1377/hlthaff.2023.00729
31	Geiger S et al.	2022	Ireland/Ireland	High-income economies (both)	Global Access to Medicines and the Legacies of Coloniality in COVID-19 Vaccine Inequity	https://www.developmenteducationreview.com/issue/issue-34/global-access-medicines-and-legacies-coloniality-covid-19-vaccine-inequity (accessed on 15 October 2025)
32	Gleeson D et al.	2023	Australia/Australia	High-income economies (both)	Global inequities in access to COVID-19 health products and technologies: A political economy analysis	10.1016/j.healthplace.2023.103051
33	Gruszczynski L et al.	2021	Hungary/Taiwan	High-income economies (both)	Between the High ideals and reality: Managing COVID-19 vaccine nationalism	10.1017/err.2021.9
34	Gupta A	2022	Switzerland (GAVI)	High-income economies (both)	COVAX can still end COVID-19 vaccine apartheid	10.1038/s41562-022-01308-8
35	Hafner M et al.	2022	UK/USA	High-income economies (both)	COVID-19 and the Cost of Vaccine Nationalism	PMC9519117
36	Hassoun N	2021	USA	High-income economies (both)	Against vaccine nationalism	10.1136/medethics-2020-107193
37	Ho CM	2023	USA	High-income economies (both)	Confronting Intellectual Property Nationalism	https://lawecommons.luc.edu/facpubs/730/ (accessed on 15 October 2025)
38	Horng DC	2024	Taiwan	High-income economies (both)	The EU’s Vaccine Diplomacy in the WHO	10.54648/eerr2024003
39	Hotez PJ	2022	USA	High-income economies (both)	COVID-19 vaccines: the imperfect instruments of vaccine diplomacy	10.1093/jtm/taac063
40	Hotez PJ et al.	2021	USA/Georgia	Mixed	Restoring Vaccine Diplomacy	10.1001/jama.2021.7439
41	Khairi LNHM et al.	2022	Malaysia/UK	Mixed	The Race for Global Equitable Access to COVID-19 Vaccines	10.3390/vaccines10081306
42	Kirgizov-Barskii AV et al.	2022	Russia/Russia	Mixed	Vaccine Diplomacy and Vaccine Nationalism	10.31278/1810-6374-2022-20-3-162-181
43	Lagman JDN	2021	Philippines	High-income economies (both)	Vaccine nationalism: a predicament in ending the COVID-19 pandemic	10.1093/pubmed/fdab088
44	Lanziotti VS et al.	2022	Brazil/Uruguay	Middle- and low-income economies (both)	Vaccine apartheid: This is not the way to end the pandemic	10.1111/jpc.15805
45	Lee I et al.	2022	UK/UK	Mixed	Global COVID-19 vaccine inequity: Preferences for overseas vaccine donations over booster shots	10.1016/j.bbih.2022.100447
46	Liu L et al.	2022	China/China	Middle- and low-income economies (both)	China’s Vaccine Diplomacy and Its Implications for Global Health Governance	10.3390/healthcare10071276
47	Mahoney R et al.	2023	USA/USA	High-income economies (both)	Global regulatory reforms to promote equitable vaccine access in the next pandemic	10.1371/journal.pgph.0002482
48	Moorthy R et al.	2022	Malaysia/Malaysia	Middle- and low-income economies (both)	A review of health security and vaccine diplomacy during COVID-19 pandemic	10.11591/ijphs.v11i2.21363
49	Moreno JD et al.	2021	Not provided	Not provided	The Vaccination Cold War	10.1002/hast.1282
50	Murhula PBB et al.	2022	South Africa/South Africa	Middle- and low-income economies (both)	The Impact of COVID-19 Vaccine Nationalism on Global Health and Human Rights to Health Standards	10.1177/0972558X221096263
51	Niu S et al.	2023	China/China	Middle- and low-income economies (both)	Beyond “Vaccine Nationalism”: China’s Cooperation with the Middle East in the COVID-19 Vaccine	https://muse.jhu.edu/pub/43/article/884132 (accessed on 15 October 2025)
52	Obinna DN	2022	USA	High-income economies (both)	Solidarity across borders: A pragmatic need for global COVID-19 vaccine equity	10.1002/hpm.3341
53	Papamichail A	2023	UK	High-income economies (both)	Reinscribing global hierarchies: COVID–19, racial capitalism and the liberal international order	10.1093/ia/iiad091
54	Paquin S et al.	2023	Canada/Canada	High-income economies (both)	The WTO and the COVID-19 “Vaccine Apartheid”: Big Pharma and the Minefield of Patents	10.17645/pag.v11i1.6177
55	Parray AA et al.	2022	Bangladesh/Bangladesh	Middle- and low-income economies (both)	Ensuring the global COVID-19 vaccine equity: Universal vaccine access strategy in the context of low and-middle-income countries	10.1080/17441692.2022.2029928
56	Peacock SJ	2022	Canada	High-income economies (both)	Vaccine nationalism will persist: global public goods need effective engagement of global citizens	10.1186/s12992-022-00802-y
57	Prasad S et al.	2022	Ukraine/Bangladesh	Middle- and low-income economies (both)	Vaccine apartheid: the separation of the world’s poorest and most vulnerable and the birth of Omicron	10.1177/25151355221107975
58	Privor-Dumm L et al.	2023	USA/Korea	High-income economies (both)	Vaccine access, equity and justice: COVID-19 vaccines and vaccination	10.1136/bmjgh-2023-011881
59	Pushkaran A et al.	2023	India/India	Middle- and low-income economies (both)	A critical analysis of COVAX alliance and corresponding global health governance and policy issues: a scoping review	10.1136/bmjgh-2023-012168
60	Riaz MMA et al.	2021	Pakistan/USA	Mixed	Global impact of vaccine nationalism during COVID-19 pandemic	10.1186/s41182-021-00394-0
61	Sabahelzain MM et al.	2021	Sudan/USA	Mixed	The politics of COVID-19 vaccine confidence	10.1016/j.coi.2021.06.007
62	Scheibner J et al.	2022	Australia/Australia	High-income economies (both)	An ethico-legal assessment of intellectual property rights and their effect on COVID-19 vaccine distribution: An Australian case study	10.1093/jlb/lsac020
63	Schuklenk U	2021	South Africa	Middle- and low-income economies (both)	Vaccine nationalism—at this point in the COVID-19 pandemic: Unjustifiable	10.1111/dewb.12332
64	Seretis SA et al.	2024	Greece/Greece	High-income economies (both)	COVID-19 Pandemic and Vaccine Imperialism	10.1177/04866134241282107
65	Shah DM et al.	2022	USA/USA	High-income economies (both)	The Impact of Neocolonialism on India’s COVID-19 Response	10.5334/aogh.3587
66	Shao Q	2024	China	Middle- and low-income economies (both)	Vaccine nationalism is not unethical from a political ethics perspective: Learning from the global COVID-19 vaccine distribution failure	10.1016/j.healthpol.2024.104996
67	Sirleaf M	2022	USA	High-income economies (both)	We Charge Vaccine Apartheid?	10.1017/jme.2023.14
68	Sparke M et al.	2022	USA/USA	High-income economies (both)	Competing Responses to Global Inequalities in Access to COVID Vaccines: Vaccine Diplomacy and Vaccine Charity Versus Vaccine Liberty	10.1093/cid/ciac361
69	Su Z et al.	2021	USA/China	Mixed	COVID-19 Vaccine Donations-Vaccine Empathy or Vaccine Diplomacy? A Narrative Literature Review	10.3390/vaccines9091024
70	Suwandi I et al.	2022	USA/USA	High-income economies (both)	COVID-19 and Imperial Value: Commodity Chains, Global Monopolies, and Catastrophe Capitalism	10.1080/21598282.2022.2093772
71	Suzuki M et al.	2023	China/China	Middle- and low-income economies (both)	Political economy of vaccine diplomacy: explaining varying strategies of China, India, and Russia’s COVID-19 vaccine diplomacy	10.1080/09692290.2022.2074514
72	Tumwine J	2021	Uganda	Middle- and low-income economies (both)	COVID-19 vaccine, apartheid and xenophobia: no need to panic!	10.4314/ahs.v21i4.1
73	Uzelac G et al.	2024	UK/Ireland	High-income economies (both)	“My Country First”: Vaccine Nationalism in England?	10.1080/13537113.2023.2285088
74	Vanderslott S et al.	2021	UK/UK	High-income economies (both)	Vaccine nationalism and internationalism: perspectives of COVID-19 vaccine trial participants in the United Kingdom	10.1136/bmjgh-2021-006305
75	Varshney S et al.	2021	India	Middle- and low-income economies (both)	Vaccine diplomacy: Exploring the benefits of international collaboration	10.5530/ctbp.2021.1.12
76	Wagner CE et al.	2021	Canada/USA	High-income economies (both)	Vaccine nationalism and the dynamics and control of SARS-CoV-2	10.1126/science.abj7364
77	Walter EM	2022	Canada	High-income economies (both)	The Jab Hurts: assessing Canada’s role in vaccine nationalism during COVID-19	10.1080/11926422.2021.2011756
78	Wei CR et al.	2023	Taiwan/Kenya	Mixed	Vaccine inequity: a threat to Africa’s recovery from COVID-19	10.1186/s41182-023-00564-2
79	Zhang D et al.	2022	The Netherlands/China	Mixed	China’s “Weaponized” Vaccine: Intertwining Between International and Domestic Politics	10.1007/s12140-021-09382-x

* According to World Bank country classifications by income level for 2024–2025. https://blogs.worldbank.org/en/opendata/world-bank-country-classifications-by-income-level-for-2024-2025. Mixed: Among the first and the last author, one’s country affiliation is high income, and the other’s is middle and low income.
