# Supplementary material for: Competing Theories on Global and Regional Vaccine Inequities: A Scoping Literature Review Within the Context of the COVID-19 Pandemic
Source: Vaccines (Basel). 2025 Dec 17;13(12):1254. doi: 10.3390/vaccines13121254 (PMC12737482; doi:10.3390/vaccines13121254)
Supplement: Supplementary file 1 [file vaccines-13-01254-s001.zip › vaccines-4007641-supplementary.pdf]

Supplementary Materials

Table S1. PubMed and Scopus search strategy

**PubMed search strategy**
("vaccine colonialism" OR "vaccine nationalism" OR "vaccine internationalism" OR "vaccine apartheid" OR "vaccine imperialism" OR "vaccine diplomacy" OR "vaccine solidarity" OR "vaccine racism”) AND ("COVID-19" OR "SARS-CoV-2" OR "COVID-19"[Mesh] OR "COVID-19 Vaccines"[Mesh]) AND (inequalities OR inequities OR disparities OR injustice OR unfairness OR "Healthcare Disparities"[Mesh] OR "Health Inequities"[Mesh]) OR ("vaccine colonialism"[Title/Abstract] OR "vaccine nationalism"[Title/Abstract] OR "vaccine internationalism"[Title/Abstract] OR "vaccine apartheid"[Title/Abstract] OR "vaccine imperialism"[Title/Abstract] OR "vaccine diplomacy"[Title/Abstract] OR "vaccine solidarity"[Title/Abstract] OR "vaccine racism"[Title/Abstract]) AND ("COVID-19"[Title/Abstract] OR "SARS-CoV2"[Title/Abstract] OR "COVID-19 vaccin*"[Title/Abstract])
**Scopus search strategy**
(ALL (("vaccine colonialism" OR "vaccine nationalism" OR "vaccine internationalism" OR "vaccine apartheid" OR "vaccine imperialism" OR "vaccine diplomacy" OR "vaccine solidarity" OR "vaccine racism") AND ("COVID-19" OR "SARS-CoV-2" OR "COVID-19 vaccine" OR "COVID-19 vaccines" OR "COVID-19 vaccination") AND (inequalities OR inequities OR disparities OR injustice OR unfairness)) OR TITLE-ABS-KEY (("vaccine colonialism" OR "vaccine nationalism" OR "vaccine internationalism" OR "vaccine apartheid" OR "vaccine imperialism" OR "vaccine diplomacy" OR "vaccine solidarity" OR "vaccine racism") AND("COVID-19" OR "SARS-CoV-2" OR "COVID-19 vaccine" OR "COVID-19 vaccines" OR "COVID-19 vaccination"))
